# OASIS-SB: a sex-balanced, distribution-preserving, synthetic phenotypic dataset for bias-resilient clinical prediction

**DOI:** 10.3389/fncom.2025.1744217

**Published:** 2026-01-16

**Authors:** Naman Dhariwal

**Affiliations:** Department of Statistics, University of Michigan, Ann Arbor, MI, United States

**Keywords:** computational neuroscience, dementia prediction, fairness in AI, neuroimaging, OASIS dataset, sex bias, SMOTE, XGBoost

## 1 Introduction

The Open Access Series of Imaging Studies (OASIS) provides one of the most widely used neuroimaging datasets for dementia and Alzheimer's disease research. The first release, OASIS-1, introduced cross-sectional T1-weighted MRI scans and clinical assessments from young, middle-aged, and older adults, both with and without dementia (Marcus et al., 2007). This dataset established a benchmark for studying brain morphology and cognitive decline across the adult lifespan. Subsequently, OASIS-2 expanded upon this framework by including longitudinal data from older adults with repeated imaging sessions, allowing researchers to examine disease progression over time (Marcus et al., 2010). The distinguishing factor between the two datasets is the presence of the “group” attribute in OASIS-2 dataset. This attribute clearly identifies each patient as dementia, non-dementia or converted, giving a key insight into the patients disease status. Together, these datasets have served as foundational resources for machine learning (ML) and deep learning studies in dementia prediction.

Over the past decade, researchers have applied a variety of computational models to OASIS data to improve diagnostic accuracy and early detection of dementia. Baglat et al. (2020) and Shrivastava et al. (2023) demonstrated that multiple ML classifiers, including Support Vector Machines, Random Forests, and Logistic Regression, can effectively distinguish people with dementia from people without dementia using OASIS-derived clinical and imaging features. Similarly, Basheer et al. (2021) employed deep neural network architectures, such as convolutional and capsule networks, achieving high predictive accuracy for Alzheimer's disease classification. Aslan and Özüpak (2025) compared a range of ML algorithms for automated Alzheimer's disease prediction, highlighting that gradient boosting and ensemble models outperform traditional classifiers in feature-rich neuroimaging datasets. Consistent with this, Shaik et al. (2025) used the OASIS dataset to predict dementia stages via deep learning, emphasizing the relevance of brain volumetric and cognitive test features in classification. Earlier, Lee and Abdullah (2019) had already demonstrated that combining neuroimaging with cognitive metrics can substantially improve predictive performance.

Despite these studies, most existing studies focus on improving accuracy or model complexity, while less attention is given to bias propagation, particularly along demographic lines such as sex. Sex-based differences in brain morphology, cognitive aging, and dementia prevalence are well established, yet many ML models trained on neuroimaging data inherit or even amplify these biases. Such biases can arise from unequal representation, data preprocessing, or label inference procedures, issues that are rarely documented or quantitatively evaluated. The OASIS dataset is primarily an imbalanced dataset with respect to sex. Both OASIS-1 and 2 contain higher number of female subjects compared to males, inducing a class-imbalance in the trained AI models.

This study constructs a unified, cross-sectional, and sex-balanced dataset by integrating the OASIS-1 and OASIS-2 studies. The longitudinal OASIS-2 data were reformatted into cross-sectional-snapshot data and used to train a predictive model that generated dementia labels for OASIS-1, after which both datasets were merged. A K-Nearest Neighbors-based Synthetic Minority Oversampling Technique (KNN-SMOTE) method was applied to correct sex imbalance, and statistical evaluation confirmed that the combined dataset retained the original sex-related patterns while expanding sample coverage for bias-aware dementia research.

This research, therefore, contributes a novel harmonized dataset OASIS-SB for dementia research and also an empirical demonstration of how preprocessing and augmentation pipelines can retain real-world sex-linked patterns despite class balancing. The dataset serves as a transparent and reproducible foundation for future studies exploring fairness, bias quantification, and equitable modeling in computational neuroscience. Importantly, OASIS-SB is derived exclusively from phenotypic and volumetric measurements extracted from OASIS and does not include MRI images; rather, it represents a statistically validated synthetic augmentation intended for cross-sectional analysis.

## 2 Methods

### 2.1 Dataset description

Two complementary subsets from the Open Access Series of Imaging Studies (OASIS) were used in this work: OASIS-1 and OASIS-2. The first dataset (Marcus et al., 2007) consists of cross-sectional magnetic resonance imaging (MRI) and clinical measures for adults spanning a wide age range, while the second (Marcus et al., 2010) contains repeated MRI sessions for older participants, enabling longitudinal observation of cognitive decline.

OASIS-2 includes explicit dementia ratings for each visit, whereas OASIS-1 does not. This structural difference restricts their combined use for statistical analysis. This study uses the OASIS-2, serving as a labeled reference for predictive model training and the OASIS-1 providing additional unlabeled samples for inference. The integration of the two datasets provides us with a significantly larger dataset with statistics similar to the real-world data. Across both datasets, key clinical and anatomical variables were retained: age, sex, education (EDUC), socioeconomic status (SES), Mini-Mental State Examination (MMSE), Clinical Dementia Rating (CDR), estimated total intracranial volume (eTIV), normalized whole-brain volume (nWBV), and atlas scaling factor (ASF).

### 2.2 Data preprocessing and target definition

To maintain consistency across both datasets, variable names and formats were standardized before merging. Continuous variables were *z*-scored, and categorical attributes such as sex were numerically encoded (female = 1, male = 0).

The longitudinal structure of OASIS-2 was flattened by treating each visit as an independent observation. Session identifiers were retained to allow optional subject-level aggregation. This transformation enabled compatibility with the single-timepoint structure of OASIS-1 and ensured that each row corresponded to one MRI-clinical snapshot.

Missing entries in SES continuous-attribute in the OASIS-2 dataset were imputed using a *Random Forest Regressor* trained on complete cases within OASIS-2. The model was optimized via five-fold cross-validation to minimize the mean absolute error (MAE) across numerical variables, achieving ROC-AUC = 0.9263. This approach preserved multivariate dependencies more effectively than mean substitution or deletion.

The clinical dementia rating (CDR) served as the ground-truth label for supervised learning. To construct a binary target suitable for classification, subjects with CDR = 0 were assigned to the class of people without dementia, and those with CDR ≠0 to the class of people with dementia. Participants labeled as Converted, those who transitioned from 0 to having dementia (CDR ≤ 0.2) between visits were excluded to avoid ambiguity in disease state. The resulting distribution produced a clean two-class binary training target.

Final predictors included demographic, cognitive, and volumetric measures [Age, Sex, EDUC, SES, MMSE, CDR, eTIV, nWBV, ASF], each normalized to unit scale prior to model fitting. This standardized preprocessing pipeline yielded a high-quality, analysis-ready dataset from OASIS-2 that could be used to train predictive models and infer dementia labels in OASIS-1.

### 2.3 Model training and label prediction

Multiple machine learning models like Random Forest, Regression models and Boosting models were tested to identify the best fit model for the OASIS-1 target prediction. After the initial study and previous studies from published mental health prediction studies (Dhariwal et al., 2024), an *Extreme Gradient Boosting* (XGBoost) classifier was trained on the preprocessed OASIS-2 dataset to predict dementia status. The model is a tree-ensemble-based framework optimized for binary logistic loss, with a learning rate of η = 0.03, a maximum depth of 3, and both subsample and column-sampling ratios set to 0.9. The XGBoost model was put through hyperparameter optimization, conducted using a grid search and five-fold stratified cross-validation. The data were divided into training (80%) and testing (20%) partitions, stratified by dementia label, and model performance was assessed using accuracy, recall, F1-score, and AUC.

Using five-fold cross-validation on the training data, the XGBoost classifier achieved a mean accuracy of 97.59% ( ± 1.55%), an F1-score of 97.31% ( ± 1.70%), and a recall of 97.02% ( ± 1.86%). When evaluated on a held-out 20% test set, the final model obtained an accuracy of 97.33%, a recall of 94.12%, an F1-score of 96.97%, and a ROC-AUC of 0.9706. Additional calibration and discrimination metrics on the test set included a PR-AUC of 0.9794.

**Figure 1:**
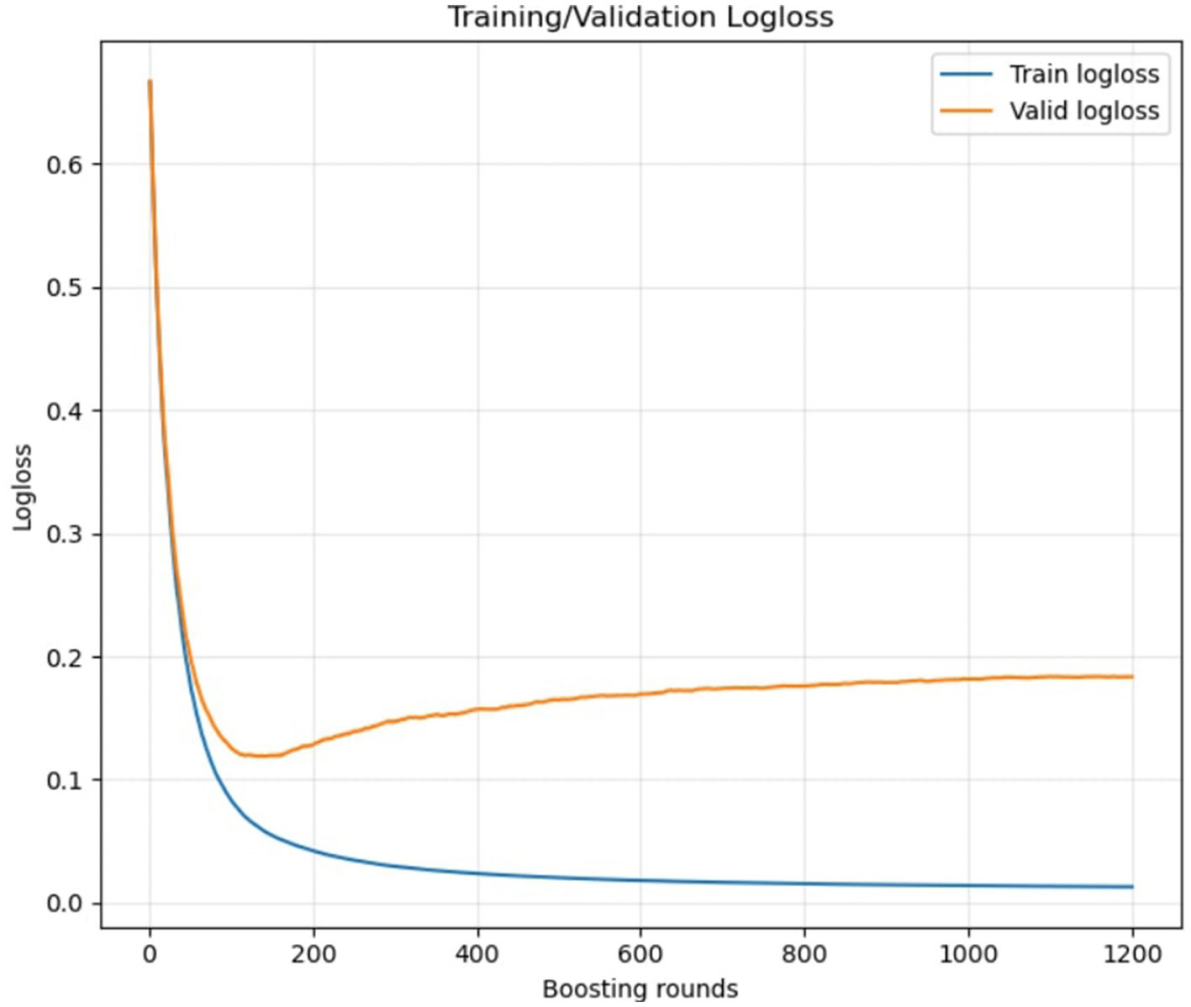
Training and validation log loss across boosting iterations showing stable model convergence.

As shown in Figure 1, the model exhibited stable convergence with minimal overfitting across training iterations. These results duly substantiate the training of the model and proves the model is a good-fit, without over or under-fitting. Feature importance scores extracted from the fitted model indicated that CDR, SES and EDUC were the top three strongest predictors of dementia classification.

The optimized hyperparameter-tuned XGBoost model was then used to predict the “group” attribute in the unlabeled OASIS-1 dataset to infer dementia status for each participant. Since, the OASIS-2 data was converted to snapshot-based cross-sectional data, the use of the trained XGBoost model to predict OASIS-1 data statistically robust. For every subject, the classifier predicted a binary prediction (*y* = 0 or 1). These inferred labels were then appended to OASIS-1 dataset, generating a complete diagnostic column “group” consistent with the OASIS-2 real-world correlations. Finally, both datasets were concatenated to form an integrated cross-sectional dataset. This dataset had 558 patient records, with a stark class-imbalance between male and female patients, having 163 more female subjects than male. This unified dataset served as the foundation for subsequent class-balancing and sex-bias evaluation procedures.

### 2.4 Class balancing by sex

A KNN-based SMOTE algorithm was applied to synthetically augment the phenotypic feature space for class balancing. The core idea at this step was to up-sample the male subjects without down-sampling the female samples and losing valuable insights. The new up-sampling was done with statistically substantiated methods like KNN-SMOTE. This algorithm generates synthetic instances for the minority class by interpolating between each male sample and its *k* = 5 nearest male neighbors in feature space, producing statistically real-world-based non-duplicated records. All numeric features were standardized prior to oversampling to ensure that distance computations remained scale-independent. The resampling process was restricted to the male subgroup, thereby achieving a 1:1 female-to-male ratio. Following resampling, basic statistical diagnostics confirmed that the means and variances of key variables (Age, MMSE, eTIV, and nWBV) remained within ± 2% of their pre-KNN-SMOTE values, indicating preservation of the dataset's core statistical structure. The resulting sex-balanced dataset was then used for subsequent correlation analyses to evaluate whether equal representation altered the intrinsic relationships between sex and other neuroanatomical or cognitive features.

## 3 Data analysis

The KNN-SMOTE balanced dataset, referred to as OASIS-SB dataset (with 712 records) was then subjected to Pearson's correlation test to evaluate whether the sex-balanced dataset follows the original statistical structure of OASIS-2. The Pearson's correlation coefficient (*r*) was used to quantify the linear association between each attribute of the dataset with the “sex” attribute and significance was assessed using two-tailed *p*-values. The correlation was specifically calculated with the “sex” column as the KNN-SMOTE sampling was applied to the sex column, therefore, the correlation analysis substantiates that the over-sampling was statistically correct. Table 1 summarizes the correlation coefficients before and after application of the KNN-SMOTE procedure.

**Table 1:** Sex-feature correlations comparison in the original OASIS-2 dataset and OASIS-SB.

**Feature**	**OASIS-2 (Pre-KNN-SMOTE)**		**OASIS-SB**	
	*r*	*p* **-value**	*r*	*p* **-value**
eTIV	–0.5719	1.08 × 10^−33^	–0.5732	1.20 × 10^−52^
ASF	0.5621	2.26 × 10^−32^	0.5629	1.86 × 10^−50^
nWBV	0.2512	9.19 × 10^−07^	0.2206	6.51 × 10^−08^
Group	–0.2479	1.30 × 10^−06^	–0.2178	9.54 × 10^−08^
CDR	–0.2106	4.24 × 10^−05^	–0.1686	3.96 × 10^−05^
MMSE	0.1668	0.00124	0.1278	0.00190
EDUC	–0.0846	0.10327	–0.1255	0.00231
SES	0.0250	0.63060	0.0333	0.42076
Age	0.0343	0.51003	0.0040	0.92210

Both the OASIS-2 and the OASIS-SB datasets show that the direction and magnitude of sex-feature correlations remained nearly identical with strong p-values. This strongly indicates that the KNN-SMOTE oversampling process retained the core underlying sex-related feature relationships. Thus, the new data with sex-balanced class (equal male and female subjects), shows strong similarity to real-world data. The strong negative associations remained consistent between sex and total intracranial volume (eTIV; *r* = −0.57) and between sex and atlas scaling factor (ASF; *r* = 0.56), uniform with known anatomical differences between male and female brain volumes. Cognitive measures such as MMSE and CDR exhibited weak but statistically significant correlations with sex in both datasets, while socioeconomic (SES) and age variables showed no significant association.

The preservation of correlation strength and significance across all key variables demonstrates that the class-balancing procedure successfully equalized representation without altering intrinsic dataset structure. This outcome confirms that the resulting OASIS-1+2 dataset maintains the original sex-linked statistical characteristics of OASIS-2, thereby retaining the natural bias present in real-world neuroimaging data while providing a larger and more balanced sample for downstream analyses.

## 4 Conclusion

This study presents a unified, cross-sectional, and sex-balanced synthetic dataset OASIS-SB derived from the OASIS-1 and OASIS-2 studies, enabling large-scale analysis of dementia prediction while maintaining transparency in bias propagation. By converting the longitudinal OASIS-2 data to a cross-sectional format, imputing missing values, and employing an XGBoost classifier to infer dementia status for OASIS-1 participants, we generated a comprehensive dataset with consistent diagnostic labeling. Subsequent KNN-SMOTE balancing achieved equal representation of male and female participants without distorting the underlying feature distributions.

Correlation analyses demonstrated that the relationships between sex and key anatomical or cognitive variables, particularly eTIV, ASF, nWBV, and CDR, remained statistically consistent with those observed in the original OASIS-2 dataset. This indicates that while the dataset was successfully balanced, the intrinsic sex-linked structure was preserved, providing a realistic foundation for fairness-aware modeling.

The resulting OASIS-SB dataset expands available cross-sectional data for dementia research and offers a transparent benchmark for studying sex bias in neuroimaging-based prediction models. Future work will extend this approach to multi-cohort harmonization and evaluate bias mitigation strategies using fairness-aware learning frameworks.

## Data Availability

The datasets presented in this study can be found in online repositories. The names of the repository/repositories and accession number(s) can be found in the article/Supplementary material.
